# Tetra­ethyl­ammonium dibromido­tricarbon­yl(*o*-toluidine)rhenate(I)

**DOI:** 10.1107/S1600536810050038

**Published:** 2010-12-04

**Authors:** Alice Brink, Hendrik G. Visser, Andreas Roodt

**Affiliations:** a Department of Chemistry, University of the Free State, PO Box 339, Bloemfontein 9300, South Africa

## Abstract

In the title compound, (C_8_H_20_N)[ReBr_2_(C_7_H_9_N)(CO)_3_], the Re^I^ atom is octa­hedrally surrounded by three carbonyl ligands orientated in a facial arrangement, two bromide ligands and an *o*-toluidine ligand. The amine lies *trans* to the carbonyl ligand and is substitutionally disordered over two positions in a 0.66 (1):0.34 (1) ratio. An array of C—H⋯O, C—H⋯Br and N—H⋯Br hydrogen-bonding inter­actions between the cations and the surrounding rhenate anions stabilize the crystal structure.

## Related literature

For the synthesis of the Re^I^–tricarbonyl synthon, see: Alberto *et al.* (1996 ▶); Brink *et al.* (2009 ▶). For related rhenium–tricarbonyl complexes, see: Mundwiler *et al.* (2004 ▶); Wang *et al.* (2003 ▶); Saw *et al.* (2006 ▶); Schutte *et al.* (2008 ▶, 2009 ▶, 2010 ▶); Wei *et al.* (2003 ▶); Schibli *et al.* (2000 ▶). For kinetic studies of related Re compounds, see: Smith *et al.* (1996 ▶); Abou-Hamdan *et al.* (1998 ▶). For related dibromido structures, see: Alberto *et al.* (1999 ▶); Abram *et al.* (1998 ▶).
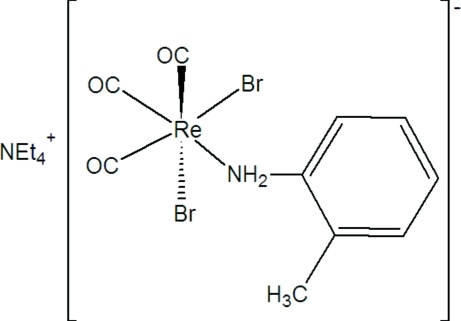


## Experimental

### 

#### Crystal data

(C_8_H_20_N)[ReBr_2_(C_7_H_9_N)(CO)_3_]
*M* *_r_* = 667.45
Monoclinic, 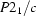

*a* = 10.776 (2) Å
*b* = 18.466 (4) Å
*c* = 11.745 (2) Å
β = 106.74 (3)°
*V* = 2238.2 (8) Å^3^
*Z* = 4
Mo *K*α radiation
μ = 9.02 mm^−1^
*T* = 100 K
0.42 × 0.32 × 0.08 mm

#### Data collection

Bruker X8 APEXII 4K Kappa CCD diffractometer
Absorption correction: multi-scan (*SADABS*; Bruker, 2004 ▶) *T*_min_ = 0.116, *T*_max_ = 0.532
45095 measured reflections
5371 independent reflections
4411 reflections with *I* > 2σ(*I*)
*R*_int_ = 0.072

#### Refinement

*R*[*F* ^2^ > 2σ(*F* ^2^)] = 0.032
*wR*(*F* ^2^) = 0.086
*S* = 1.06
5371 reflections
270 parameters
H-atom parameters constrained
Δρ_max_ = 2.5 e Å^−3^
Δρ_min_ = −2.99 e Å^−3^

### 

Data collection: *APEX2* (Bruker, 2005 ▶); cell refinement: *SAINT-Plus* (Bruker, 2004 ▶); data reduction: *SAINT-Plus* and *XPREP* (Bruker, 2004 ▶); program(s) used to solve structure: *SIR92* (Altomare *et al.*, 1999 ▶); program(s) used to refine structure: *SHELXL97* (Sheldrick, 2008 ▶); molecular graphics: *DIAMOND* (Brandenburg & Putz, 2004 ▶); software used to prepare material for publication: *WinGX* (Farrugia, 1999 ▶).

## Supplementary Material

Crystal structure: contains datablocks global, I. DOI: 10.1107/S1600536810050038/bt5421sup1.cif

Structure factors: contains datablocks I. DOI: 10.1107/S1600536810050038/bt5421Isup2.hkl

Additional supplementary materials:  crystallographic information; 3D view; checkCIF report

## Figures and Tables

**Table 1:** Hydrogen-bond geometry (Å, °)

*D*—H⋯*A*	*D*—H	H⋯*A*	*D*⋯*A*	*D*—H⋯*A*
N1—H1*C*⋯Br2^i^	0.92	2.7	3.542 (4)	153
N1—H1*B*⋯Br1^i^	0.92	2.75	3.594 (4)	153
C121—H12*C*⋯O03^ii^	0.98	2.5	3.139 (10)	123
C35—H35*B*⋯O03^iii^	0.99	2.39	3.196 (7)	138
C37—H37*A*⋯Br1^iv^	0.99	2.92	3.911 (6)	174

Symmetry codes: (i) 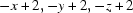; (ii) 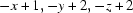; (iii) 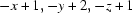; (iv) 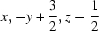.

**Fig. 1.**
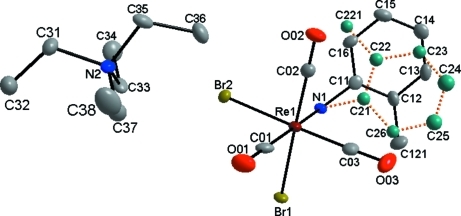
Representation of the molecular structure of the title compound, showing the numbering scheme and displacement ellipsoids drawn at 50% probability level. Hydrogen atoms are omitted for clarity.

**Fig. 2.**
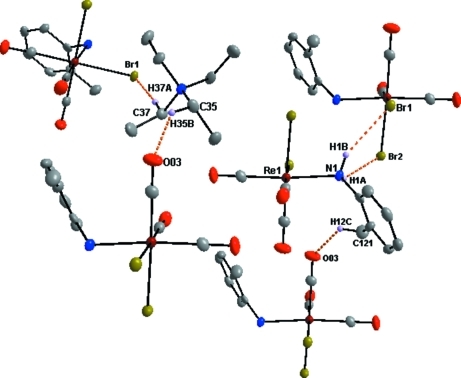
Representation of the hydrogen-bonding interactions (only one complete molecular structure (symm. op.: x, y, z) is shown).

## supplementary crystallographic information

### Comment

The structure forms part of an ongoing investigation aimed at determining the structural and kinetic behaviour of *fac*-rhenium tricarbonyl complexes. Various rhenium bi- and tridentate tricarbonyl ligands have been synthesized (Mundwiler *et al.*, 2004, Wang *et al.*, 2003, Saw *et al.*, 2006, Schutte *et al.*, 2009, 2008, 2010, Wei *et al.*, 2003, Schibli *et al.*, 2000). A few crystallographic studies on dibromido monodentate rhenium compounds have been reported in literature (Alberto *et al.*, 1999, Abram *et al.*, 1998).

The title complex crystallized as a distorted octahedral anionic Re^I^ compound with one tetraethylammonium counter ion in the asymmetric unit (Fig. 1). The coordinated amine lies in an axial position below the equatorial plane, defined as Br1—Br2—C02—C03, and *trans* to a carbonyl ligand. It is disordered over two positions and the plane through the aromatic carbons lies at an angle of 35.2 (2)° to the equatorial plane. The Re—N bond distance (2.241 (4) Å) is longer than for the rhenium acetonitrile analogue (2.150 (6) Å) (Abram *et al.*, 1998).

The longer Re—Br bond lengths (2.6390 (7) Å and 2.6370 (8) Å) are induced by the facially coordinated carbonyl ligands and compares well with related structures (Abram *et al.*, 1998, Schutte *et al.*, 2010). Intermolecular C—H···O, C—H···Br and N—H···Br hydrogen-bonding interactions are observed between rhenate anions and neighboring cations.

### Experimental

[NEt_4_]_2_[Re(CO)_3_Br_3_] (0.13 mmol) (synthesized according to Alberto *et al.* (1996)) was dissolved in 6 ml methanol. The ligand 2-(*o*-tolyliminomethyl)phenol (0.14 mmol) (for related synthesis see Brink *et al.*, 2009), containing 10% *o*-toluidine as byproduct, was dissolved in 6 ml MeOH and slowly added. The reaction mixture was stirred for 2 h at room temperature. Crystals of the title complex whereby the Re bonded preferentially to the amine were obtained by the slow evaporation of the solvent at 4°C.

### Refinement

The aromatic and aliphatic H atoms were placed in geometrically idealized positions and constrained to ride on their parent atoms with *U*_iso_(H) = 1.2*U*_eq_(C) or *U*_iso_(H) = 1.5_eq_(C_methyl_). The methyl groups were generated to fit the difference electron density and the groups were then refined as rigid rotors. The highest peak in the final difference map are located 0.81Å from Re1. The minor occupied atoms were refined isotropically.

### Crystal data

**Table:**

(C_8_H_20_N)[ReBr_2_(C_7_H_9_N)(CO)_3_]	*F*(000) = 1280
*M**_r_* = 667.45	*D*_x_ = 1.981 Mg m^−^^3^
Monoclinic, *P*2_1_/*c*	Mo *K*α radiation, λ = 0.71073 Å
Hall symbol: -P 2ybc	Cell parameters from 9885 reflections
*a* = 10.776 (2) Å	θ = 3.2–28.3°
*b* = 18.466 (4) Å	µ = 9.02 mm^−^^1^
*c* = 11.745 (2) Å	*T* = 100 K
β = 106.74 (3)°	Plate, yellow
*V* = 2238.2 (8) Å^3^	0.42 × 0.32 × 0.08 mm
*Z* = 4	

### Data collection

**Table:**

Bruker X8 APEXII 4K Kappa CCD diffractometer	5371 independent reflections
Radiation source: sealed tube	4411 reflections with *I* > 2σ(*I*)
graphite	*R*_int_ = 0.072
Detector resolution: 512 pixels mm^-1^	θ_max_ = 28°, θ_min_ = 3.2°
ω and φ scans	*h* = −14→13
Absorption correction: multi-scan (*SADABS*; Bruker, 2004)	*k* = −24→24
*T*_min_ = 0.116, *T*_max_ = 0.532	*l* = −15→15
45095 measured reflections	

### Refinement

**Table:**

Refinement on *F*^2^	0 restraints
Least-squares matrix: full	H-atom parameters constrained
*R*[*F*^2^ > 2σ(*F*^2^)] = 0.032	*w* = 1/[σ^2^(*F*_o_^2^) + (0.0446*P*)^2^ + 2.6175*P*] where *P* = (*F*_o_^2^ + 2*F*_c_^2^)/3
*wR*(*F*^2^) = 0.086	(Δ/σ)_max_ = 0.001
*S* = 1.06	Δρ_max_ = 2.5 e Å^−^^3^
5371 reflections	Δρ_min_ = −2.99 e Å^−^^3^
270 parameters	

### Special details

**Table:**

Experimental. The intensity data was collected on a Bruker X8 Apex II 4 K Kappa CCD diffractometer using an exposure time of 30 s/frame. A total of 1977 frames were collected with a frame width of 0.5° covering up to θ = 28.0° with 99.4% completeness accomplished
Geometry. All e.s.d.'s (except the e.s.d. in the dihedral angle between two l.s. planes) are estimated using the full covariance matrix. The cell e.s.d.'s are taken into account individually in the estimation of e.s.d.'s in distances, angles and torsion angles; correlations between e.s.d.'s in cell parameters are only used when they are defined by crystal symmetry. An approximate (isotropic) treatment of cell e.s.d.'s is used for estimating e.s.d.'s involving l.s. planes.

### Fractional atomic coordinates and isotropic or equivalent isotropic displacement parameters (Å^2^)

**Table:**

	*x*	*y*	*z*	*U*_iso_*/*U*_eq_	Occ. (<1)
Re1	0.735442 (18)	0.985810 (11)	0.808028 (15)	0.01480 (7)	
Br1	0.81038 (5)	0.89501 (3)	0.98836 (4)	0.01871 (12)	
Br2	0.95834 (5)	0.96560 (3)	0.76184 (4)	0.01895 (12)	
N1	0.8495 (4)	1.0651 (2)	0.9422 (3)	0.0174 (9)	
H1A	0.8563	1.0468	1.0166	0.021*	0.659 (10)
H1B	0.932	1.0671	0.9343	0.021*	0.659 (10)
H1C	0.888	1.0409	1.0119	0.021*	0.341 (10)
H1D	0.9145	1.0842	0.9148	0.021*	0.341 (10)
N2	0.8171 (4)	0.8488 (2)	0.3722 (3)	0.0165 (8)	
O01	0.6021 (4)	0.8662 (2)	0.6363 (3)	0.0324 (10)	
O02	0.6462 (4)	1.0930 (2)	0.6027 (3)	0.0294 (9)	
O03	0.4809 (4)	1.0153 (2)	0.8640 (4)	0.0363 (11)	
C01	0.6523 (5)	0.9121 (3)	0.7010 (4)	0.0208 (11)	
C02	0.6823 (5)	1.0544 (3)	0.6816 (4)	0.0211 (11)	
C03	0.5790 (5)	1.0036 (3)	0.8445 (5)	0.0227 (11)	
C11	0.8014 (8)	1.1388 (5)	0.9379 (8)	0.0200 (19)	0.659 (10)
C12	0.7270 (8)	1.1603 (5)	1.0104 (7)	0.022 (2)	0.659 (10)
C13	0.6778 (8)	1.2305 (5)	0.9964 (9)	0.025 (2)	0.659 (10)
H13	0.627	1.2465	1.0456	0.03*	0.659 (10)
C14	0.7005 (8)	1.2773 (6)	0.9139 (8)	0.025 (2)	0.659 (10)
H14	0.6648	1.3247	0.9058	0.03*	0.659 (10)
C15	0.7754 (8)	1.2551 (5)	0.8425 (7)	0.025 (2)	0.659 (10)
H15	0.7924	1.2871	0.7854	0.03*	0.659 (10)
C16	0.8250 (9)	1.1859 (6)	0.8557 (8)	0.023 (2)	0.659 (10)
H16	0.8766	1.1703	0.807	0.027*	0.659 (10)
C121	0.7031 (9)	1.1098 (5)	1.1036 (9)	0.032 (2)	0.659 (10)
H12A	0.638	1.1311	1.1371	0.048*	0.659 (10)
H12B	0.7841	1.1025	1.167	0.048*	0.659 (10)
H12C	0.6717	1.0631	1.0669	0.048*	0.659 (10)
C25	0.6163 (16)	1.1668 (10)	1.0700 (14)	0.029 (4)*	0.341 (10)
H25	0.5649	1.1593	1.1224	0.035*	0.341 (10)
C22	0.7679 (15)	1.1900 (9)	0.9138 (13)	0.021 (4)*	0.341 (10)
C24	0.615 (2)	1.2320 (11)	1.0160 (16)	0.032 (4)*	0.341 (10)
H24	0.5629	1.2698	1.0328	0.039*	0.341 (10)
C23	0.6844 (18)	1.2448 (12)	0.9392 (19)	0.020 (4)*	0.341 (10)
H23	0.6782	1.2906	0.9013	0.025*	0.341 (10)
C21	0.7704 (18)	1.1241 (11)	0.9674 (16)	0.020 (5)*	0.341 (10)
C26	0.6933 (16)	1.1111 (10)	1.0483 (16)	0.019 (4)*	0.341 (10)
H26	0.6955	1.0653	1.0859	0.023*	0.341 (10)
C221	0.8433 (19)	1.2027 (11)	0.8293 (17)	0.018 (5)*	0.341 (10)
H22A	0.8303	1.2526	0.8002	0.027*	0.341 (10)
H22B	0.8145	1.1692	0.7622	0.027*	0.341 (10)
H22C	0.9355	1.1947	0.8694	0.027*	0.341 (10)
C31	0.8711 (5)	0.8176 (3)	0.2768 (4)	0.0240 (11)	
H31A	0.8	0.8148	0.2015	0.029*	
H31B	0.937	0.8514	0.2639	0.029*	
C32	0.9320 (6)	0.7433 (3)	0.3040 (5)	0.0321 (14)	
H32A	0.9594	0.7263	0.2359	0.048*	
H32B	0.8686	0.7095	0.3192	0.048*	
H32C	1.0075	0.7462	0.3743	0.048*	
C33	0.9209 (5)	0.8507 (3)	0.4913 (4)	0.0215 (11)	
H33A	0.8832	0.8725	0.5508	0.026*	
H33B	0.9455	0.8003	0.5165	0.026*	
C34	1.0420 (5)	0.8920 (4)	0.4925 (5)	0.0336 (14)	
H34A	1.0957	0.8983	0.5748	0.05*	
H34B	1.0181	0.9396	0.4558	0.05*	
H34C	1.0907	0.865	0.4478	0.05*	
C35	0.7702 (5)	0.9248 (3)	0.3309 (4)	0.0217 (11)	
H35A	0.8448	0.9532	0.3222	0.026*	
H35B	0.7065	0.9212	0.2513	0.026*	
C36	0.7084 (6)	0.9660 (3)	0.4118 (5)	0.0281 (12)	
H36A	0.6865	1.0151	0.3807	0.042*	
H36B	0.7693	0.9687	0.4918	0.042*	
H36C	0.6294	0.941	0.4153	0.042*	
C37	0.7062 (5)	0.8034 (3)	0.3888 (5)	0.0249 (12)	
H37A	0.7392	0.7541	0.4133	0.03*	
H37B	0.6777	0.8244	0.4546	0.03*	
C38	0.5894 (6)	0.7972 (3)	0.2804 (6)	0.0370 (15)	
H38A	0.5259	0.7642	0.2977	0.056*	
H38B	0.6167	0.7782	0.2134	0.056*	
H38C	0.5502	0.8451	0.2599	0.056*	

### Atomic displacement parameters (Å^2^)

**Table:**

	*U*^11^	*U*^22^	*U*^33^	*U*^12^	*U*^13^	*U*^23^
Re1	0.01788 (12)	0.01279 (12)	0.01337 (10)	−0.00031 (8)	0.00393 (8)	0.00151 (7)
Br1	0.0253 (3)	0.0146 (2)	0.0156 (2)	−0.0011 (2)	0.00488 (19)	0.00306 (17)
Br2	0.0216 (3)	0.0210 (3)	0.0157 (2)	−0.0011 (2)	0.00766 (19)	−0.00174 (18)
N1	0.023 (2)	0.012 (2)	0.0162 (19)	−0.0010 (17)	0.0056 (16)	0.0001 (15)
N2	0.021 (2)	0.015 (2)	0.0133 (18)	0.0006 (17)	0.0048 (16)	0.0014 (15)
O01	0.041 (2)	0.025 (2)	0.026 (2)	−0.0135 (19)	0.0027 (17)	−0.0051 (17)
O02	0.044 (2)	0.020 (2)	0.0220 (19)	0.0010 (18)	0.0058 (17)	0.0058 (16)
O03	0.025 (2)	0.055 (3)	0.033 (2)	0.008 (2)	0.0136 (18)	0.014 (2)
C01	0.023 (3)	0.018 (3)	0.020 (2)	−0.004 (2)	0.005 (2)	0.008 (2)
C02	0.025 (3)	0.020 (3)	0.019 (2)	−0.001 (2)	0.008 (2)	−0.001 (2)
C03	0.024 (3)	0.022 (3)	0.021 (3)	0.000 (2)	0.006 (2)	0.007 (2)
C11	0.012 (4)	0.027 (5)	0.017 (4)	−0.004 (4)	−0.002 (3)	−0.003 (3)
C12	0.023 (4)	0.020 (4)	0.022 (4)	0.004 (3)	0.005 (3)	−0.005 (3)
C13	0.018 (5)	0.028 (5)	0.029 (5)	0.001 (4)	0.008 (4)	−0.008 (4)
C14	0.023 (5)	0.022 (5)	0.026 (4)	−0.002 (4)	0.001 (3)	−0.004 (4)
C15	0.023 (4)	0.022 (5)	0.026 (4)	−0.006 (4)	−0.002 (3)	0.006 (3)
C16	0.027 (5)	0.028 (5)	0.016 (4)	−0.004 (4)	0.009 (4)	0.000 (4)
C121	0.039 (6)	0.035 (6)	0.026 (5)	−0.006 (4)	0.016 (4)	0.001 (4)
C31	0.033 (3)	0.025 (3)	0.016 (2)	0.003 (2)	0.011 (2)	−0.001 (2)
C32	0.050 (4)	0.022 (3)	0.029 (3)	0.000 (3)	0.019 (3)	−0.004 (2)
C33	0.031 (3)	0.018 (3)	0.012 (2)	0.008 (2)	0.001 (2)	0.0021 (18)
C34	0.023 (3)	0.041 (4)	0.032 (3)	0.001 (3)	0.001 (2)	−0.004 (3)
C35	0.023 (3)	0.015 (3)	0.023 (2)	0.000 (2)	0.001 (2)	0.006 (2)
C36	0.033 (3)	0.020 (3)	0.030 (3)	0.007 (2)	0.007 (2)	−0.003 (2)
C37	0.027 (3)	0.020 (3)	0.031 (3)	−0.004 (2)	0.013 (2)	−0.003 (2)
C38	0.028 (3)	0.029 (4)	0.052 (4)	−0.010 (3)	0.008 (3)	−0.011 (3)

### Geometric parameters (Å, °)

**Table:**

Re1—C03	1.884 (6)	C22—C21	1.37 (2)
Re1—C01	1.895 (5)	C22—C23	1.44 (2)
Re1—C02	1.909 (5)	C22—C221	1.47 (2)
Re1—N1	2.241 (4)	C24—C23	1.35 (3)
Re1—Br2	2.6370 (8)	C24—H24	0.95
Re1—Br1	2.6389 (7)	C23—H23	0.95
N1—C11	1.452 (10)	C21—C26	1.45 (3)
N1—C21	1.47 (2)	C26—H26	0.95
N1—H1A	0.92	C221—H22A	0.98
N1—H1B	0.92	C221—H22B	0.98
N1—H1C	0.92	C221—H22C	0.98
N1—H1D	0.92	C31—C32	1.514 (8)
N2—C31	1.518 (6)	C31—H31A	0.99
N2—C37	1.518 (6)	C31—H31B	0.99
N2—C33	1.518 (6)	C32—H32A	0.98
N2—C35	1.523 (6)	C32—H32B	0.98
O01—C01	1.162 (6)	C32—H32C	0.98
O02—C02	1.145 (6)	C33—C34	1.508 (8)
O03—C03	1.164 (7)	C33—H33A	0.99
C11—C16	1.377 (14)	C33—H33B	0.99
C11—C12	1.385 (12)	C34—H34A	0.98
C12—C13	1.392 (12)	C34—H34B	0.98
C12—C121	1.516 (12)	C34—H34C	0.98
C13—C14	1.372 (13)	C35—C36	1.513 (7)
C13—H13	0.95	C35—H35A	0.99
C14—C15	1.383 (12)	C35—H35B	0.99
C14—H14	0.95	C36—H36A	0.98
C15—C16	1.377 (13)	C36—H36B	0.98
C15—H15	0.95	C36—H36C	0.98
C16—H16	0.95	C37—C38	1.515 (7)
C121—H12A	0.98	C37—H37A	0.99
C121—H12B	0.98	C37—H37B	0.99
C121—H12C	0.98	C38—H38A	0.98
C25—C24	1.36 (3)	C38—H38B	0.98
C25—C26	1.39 (2)	C38—H38C	0.98
C25—H25	0.95		
			
C03—Re1—C01	89.6 (2)	C23—C24—C25	122 (2)
C03—Re1—C02	88.5 (2)	C23—C24—H24	118.9
C01—Re1—C02	89.0 (2)	C25—C24—H24	118.9
C03—Re1—N1	94.1 (2)	C24—C23—C22	121 (2)
C01—Re1—N1	174.31 (19)	C24—C23—H23	119.5
C02—Re1—N1	95.41 (19)	C22—C23—H23	119.5
C03—Re1—Br2	177.68 (17)	C22—C21—C26	120.7 (17)
C01—Re1—Br2	92.67 (16)	C22—C21—N1	120.3 (15)
C02—Re1—Br2	91.17 (16)	C26—C21—N1	119.0 (15)
N1—Re1—Br2	83.64 (11)	C25—C26—C21	118.8 (17)
C03—Re1—Br1	90.87 (16)	C25—C26—H26	120.6
C01—Re1—Br1	93.02 (15)	C21—C26—H26	120.6
C02—Re1—Br1	177.91 (15)	C22—C221—H22A	109.5
N1—Re1—Br1	82.64 (11)	C22—C221—H22B	109.5
Br2—Re1—Br1	89.37 (3)	H22A—C221—H22B	109.5
C11—N1—Re1	118.0 (4)	C22—C221—H22C	109.5
C21—N1—Re1	113.2 (8)	H22A—C221—H22C	109.5
C11—N1—H1A	107.8	H22B—C221—H22C	109.5
C21—N1—H1A	88.4	C32—C31—N2	115.1 (4)
Re1—N1—H1A	107.8	C32—C31—H31A	108.5
C11—N1—H1B	107.8	N2—C31—H31A	108.5
C21—N1—H1B	128.7	C32—C31—H31B	108.5
Re1—N1—H1B	107.8	N2—C31—H31B	108.5
H1A—N1—H1B	107.1	H31A—C31—H31B	107.5
C11—N1—H1C	123.4	C31—C32—H32A	109.5
C21—N1—H1C	108.7	C31—C32—H32B	109.5
Re1—N1—H1C	108.9	H32A—C32—H32B	109.5
H1B—N1—H1C	84.8	C31—C32—H32C	109.5
C11—N1—H1D	86	H32A—C32—H32C	109.5
C21—N1—H1D	109.3	H32B—C32—H32C	109.5
Re1—N1—H1D	108.9	C34—C33—N2	115.2 (4)
H1A—N1—H1D	127.7	C34—C33—H33A	108.5
H1C—N1—H1D	107.7	N2—C33—H33A	108.5
C31—N2—C37	111.6 (4)	C34—C33—H33B	108.5
C31—N2—C33	110.6 (4)	N2—C33—H33B	108.5
C37—N2—C33	107.0 (4)	H33A—C33—H33B	107.5
C31—N2—C35	106.1 (4)	C33—C34—H34A	109.5
C37—N2—C35	110.4 (4)	C33—C34—H34B	109.5
C33—N2—C35	111.2 (4)	H34A—C34—H34B	109.5
O01—C01—Re1	179.1 (5)	C33—C34—H34C	109.5
O02—C02—Re1	176.6 (5)	H34A—C34—H34C	109.5
O03—C03—Re1	178.2 (5)	H34B—C34—H34C	109.5
C16—C11—C12	120.4 (9)	C36—C35—N2	115.4 (4)
C16—C11—N1	118.6 (8)	C36—C35—H35A	108.4
C12—C11—N1	120.9 (8)	N2—C35—H35A	108.4
C11—C12—C13	117.6 (9)	C36—C35—H35B	108.4
C11—C12—C121	121.0 (8)	N2—C35—H35B	108.4
C13—C12—C121	121.3 (8)	H35A—C35—H35B	107.5
C14—C13—C12	122.0 (9)	C35—C36—H36A	109.5
C14—C13—H13	119	C35—C36—H36B	109.5
C12—C13—H13	119	H36A—C36—H36B	109.5
C13—C14—C15	119.8 (9)	C35—C36—H36C	109.5
C13—C14—H14	120.1	H36A—C36—H36C	109.5
C15—C14—H14	120.1	H36B—C36—H36C	109.5
C16—C15—C14	118.8 (8)	C38—C37—N2	115.4 (4)
C16—C15—H15	120.6	C38—C37—H37A	108.4
C14—C15—H15	120.6	N2—C37—H37A	108.4
C11—C16—C15	121.4 (9)	C38—C37—H37B	108.4
C11—C16—H16	119.3	N2—C37—H37B	108.4
C15—C16—H16	119.3	H37A—C37—H37B	107.5
C24—C25—C26	119.9 (17)	C37—C38—H38A	109.5
C24—C25—H25	120.1	C37—C38—H38B	109.5
C26—C25—H25	120.1	H38A—C38—H38B	109.5
C21—C22—C23	117.4 (17)	C37—C38—H38C	109.5
C21—C22—C221	120.7 (16)	H38A—C38—H38C	109.5
C23—C22—C221	121.8 (17)	H38B—C38—H38C	109.5
			
C03—Re1—N1—C11	−53.5 (5)	C21—C22—C23—C24	2(3)
C02—Re1—N1—C11	35.4 (5)	C221—C22—C23—C24	179.3 (18)
Br2—Re1—N1—C11	126.0 (5)	C23—C22—C21—C26	−1(2)
Br1—Re1—N1—C11	−143.8 (5)	C221—C22—C21—C26	−178.4 (16)
C03—Re1—N1—C21	−27.2 (8)	C23—C22—C21—N1	177.8 (14)
C02—Re1—N1—C21	61.6 (8)	C221—C22—C21—N1	1(2)
Br2—Re1—N1—C21	152.2 (8)	C11—N1—C21—C22	8.0 (12)
Br1—Re1—N1—C21	−117.6 (8)	Re1—N1—C21—C22	−99.0 (15)
C21—N1—C11—C16	−165 (2)	C11—N1—C21—C26	−173 (3)
Re1—N1—C11—C16	−80.3 (8)	Re1—N1—C21—C26	79.9 (15)
C21—N1—C11—C12	11.7 (19)	C24—C25—C26—C21	0(3)
Re1—N1—C11—C12	96.2 (7)	C22—C21—C26—C25	0(3)
C16—C11—C12—C13	0.2 (12)	N1—C21—C26—C25	−178.6 (14)
N1—C11—C12—C13	−176.2 (7)	C37—N2—C31—C32	−63.7 (6)
C16—C11—C12—C121	−178.4 (8)	C33—N2—C31—C32	55.3 (6)
N1—C11—C12—C121	5.2 (12)	C35—N2—C31—C32	176.0 (5)
C11—C12—C13—C14	0.4 (12)	C31—N2—C33—C34	56.7 (6)
C121—C12—C13—C14	178.9 (8)	C37—N2—C33—C34	178.4 (5)
C12—C13—C14—C15	−0.7 (13)	C35—N2—C33—C34	−60.9 (6)
C13—C14—C15—C16	0.5 (13)	C31—N2—C35—C36	178.4 (4)
C12—C11—C16—C15	−0.4 (13)	C37—N2—C35—C36	57.3 (6)
N1—C11—C16—C15	176.1 (7)	C33—N2—C35—C36	−61.3 (6)
C14—C15—C16—C11	0.0 (13)	C31—N2—C37—C38	−61.2 (6)
C26—C25—C24—C23	1(3)	C33—N2—C37—C38	177.7 (5)
C25—C24—C23—C22	−2(3)	C35—N2—C37—C38	56.6 (6)

### Hydrogen-bond geometry (Å, °)

**Table:**

*D*—H···*A*	*D*—H	H···*A*	*D*···*A*	*D*—H···*A*
N1—H1C···Br2^i^	0.92	2.7	3.542 (4)	153.
N1—H1B···Br1^i^	0.92	2.75	3.594 (4)	153.
C121—H12C···O03^ii^	0.98	2.5	3.139 (10)	123
C35—H35B···O03^iii^	0.99	2.39	3.196 (7)	138.
C37—H37A···Br1^iv^	0.99	2.92	3.911 (6)	174.

Symmetry codes: (i) −*x*+2, −*y*+2, −*z*+2; (ii) −*x*+1, −*y*+2, −*z*+2; (iii) −*x*+1, −*y*+2, −*z*+1; (iv) *x*, −*y*+3/2, *z*−1/2.
